# Supplementary material for: Caveolin-1 Down-Regulation Reduces VEGF-A Secretion Induced by IGF-1 in ARPE-19 Cells
Source: Life (Basel). 2021 Dec 28;12(1):44. doi: 10.3390/life12010044 (PMC8781830; doi:10.3390/life12010044)
Supplement: Supplementary file 1 [file life-12-00044-s001.zip › life-1456573-supplementary.pdf]

Figure S1a and S1b

Membrane has been cut at the level of 70 kDa and 35 kDa: upper fragment has been immunoblotted with anti-IGF1Rβ, the middle fragment with β-actin, and the bottom with anti-Cav-1 antibodies. Images of IGF1Rβ and cav-1 have been captured from the same membrane setting, but with different exposure time.

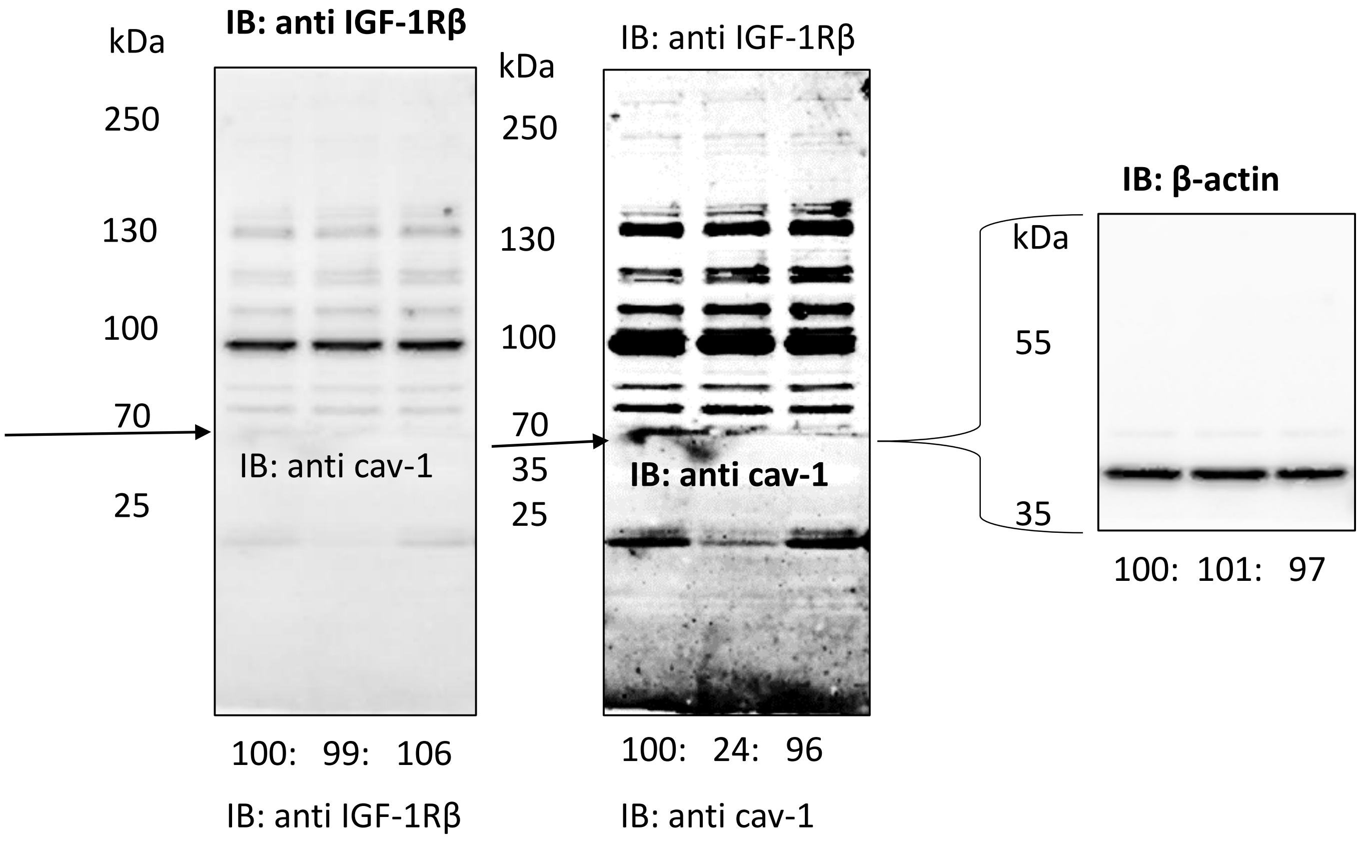

Densitometric analysis/intensity ratio

	IGF1R/ACT	CAV-1/ACT
CTR	0,59133076	0,7260386
Si-Cav-1	0,58524315	0,1754099
Si-CTR	0,62836111	0,6949462

Figure S1c and S1d

IB: anti AKT: membrane has been cut at the level of 100 kDa. The upper fragment has not been used. Membrane was strepped and reprobed with anti β-Actin.

IB: anti ERK1/2: membrane has been cut between 70 kDa and 55 KDa. The upper fragment has not been used. Membrane was strepped and reprobed with anti β-Actin.

kDa IB: anti Akt

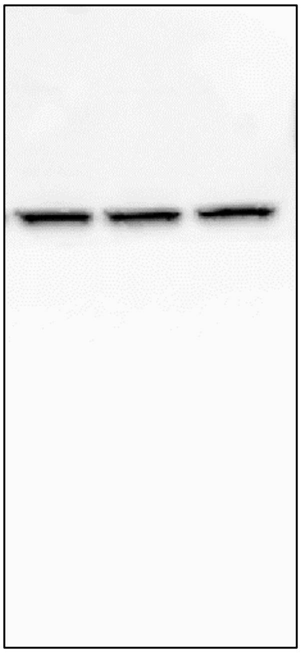

100: 103: 101

kDa IB: anti ERK 1/2

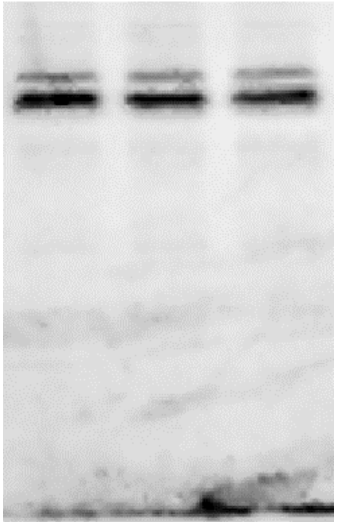

100: 98: 95

kDa IB: anti β-Actin

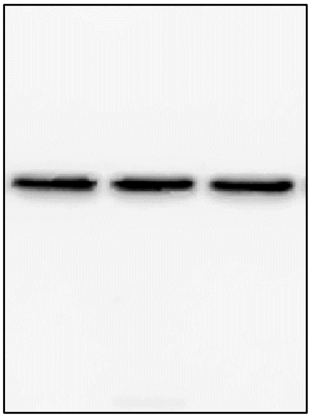

IB: anti β-Actin

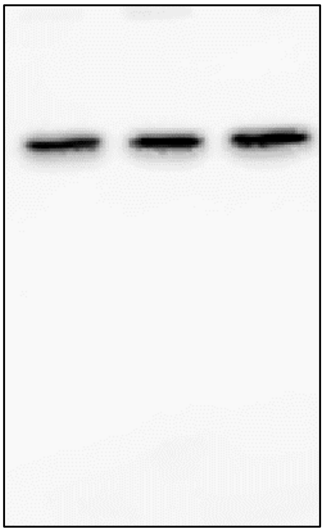

Densitometric analysis/intensity ratio

	AKT/ACT
CTR	0,957371
Si-Cav-1	0,986696
Si-CTR	0,966248

	ERK/ACT
CTR	0,984099
Si-Cav-1	0,962864
Si-CTR	0,937497

Figure S2a

IB: anti phosphoIGF-1Rβ: membrane has been cut at the level of 130 kDa. The membrane has been stripped, however the band at 70 kDa was already visible. The upper and the lower fragments have not been used.

IB: anti phosphoIGF-1Rβ

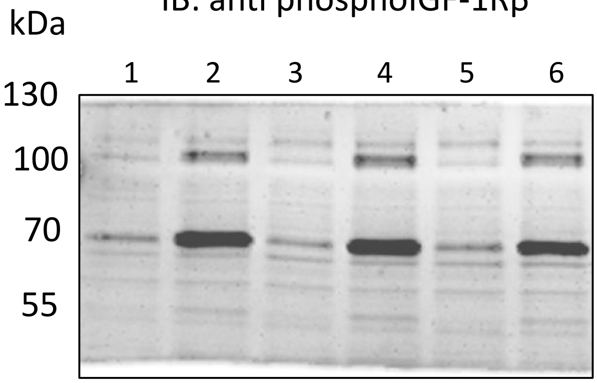

100: 678 : 107: 699: 96 : 686

IB: anti IGF-1Rβ: membrane has been cut at the level of 70 kDa. The lower fragments for actin detection.

IB: anti IGF-1Rβ

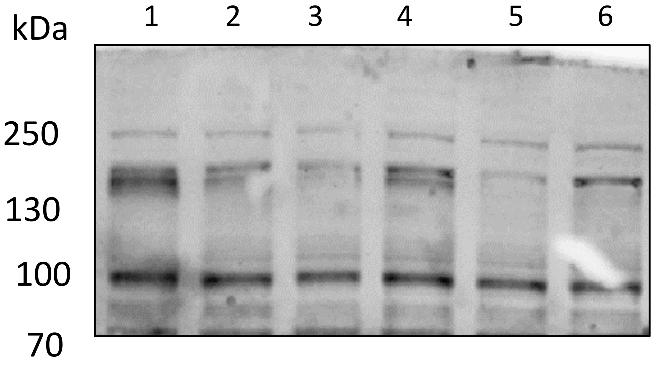

100 : 98 : 91 : 99 : 96 : 95

Densitometric analysis/intensity ratio

	phIGF1R/ACT
1	0,373098
2	2,52871
3	0,400523
4	2,609419
5	0,359456
6	2,558247

	IGF1R/ACT
1	0,774617
2	0,759392
3	0,704847
4	0,766441
5	0,740758
6	0,73419

Figure S2b

IB: anti ph-AKT: membrane has been cut at the level of 100 kDa. The upper fragment has not been used.

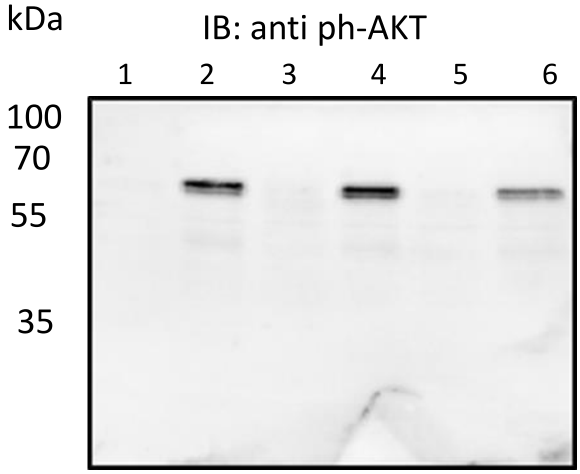

100 : 809 : 89 : 1385 : 86 : 745

IB: anti AKT: membrane has been cut between 70 kDa and 55 KDa. The other fragment has been used for other experiments.

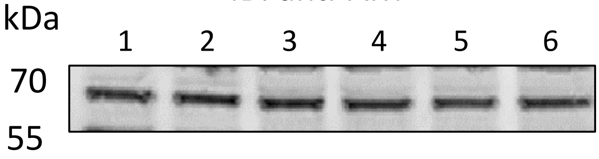

100 : 99 : 104 : 104 : 94 : 101

Densitometric analysis/intensity ratio

	phAKT/ACT
1	0,043937
2	0,355659
3	0,039284
4	0,608671
5	0,038006
6	0,327253

	AKT/ACT
1	1,107822
2	1,092836
3	1,149699
4	1,147532
5	1,039049
6	1,116509

Figure S2c

IB: anti ph-ERK1/2: membrane has been cut at the between 70 kDa and 55 kDa, and at the level of 35 kDa. The other fragments have been used for another experiment

IB: anti ph-ERK1/2

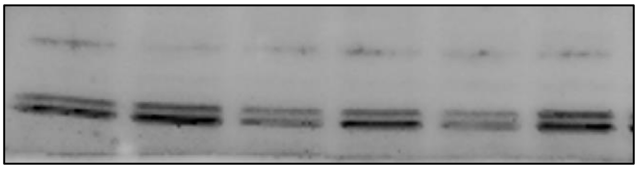

100 : 230 : 112 : 199 : 101 : 274

IB: anti ph-ERK1/2: membrane has been cut at the level of 55 kDa. The upper fragments have been used for another experiment.

IB: anti ERK1/2

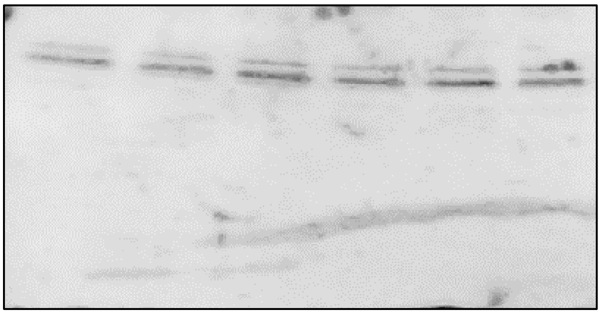

100 : 98 : 107 : 103 : 105 : 102

Densitometric analysis/intensity ratio

	phERK/ACT
1	0,866257
2	1,993088
3	0,970045
4	1,725321
5	0,874852
6	2,375852

	ERK/ACT
1	0,40533
2	0,397458
3	0,435652
4	0,416379
5	0,424357
6	0,414359
